# London Letter

**Published:** 1881-10

**Authors:** W. T. Belfield

**Affiliations:** London


### ARTICLE XII.

LONDON, Aug. 9, 1881.

EDITORS CHICAGO MEDICAL JOURNAL AND EXAMINER:—Although the present Congress will scarcely constitute an "epoch in the history of medicine," as the more enthusiastic affirm, it has been the most notable, as well as the largest gathering of medical men that the world has ever seen. Everything has conduced to the success of the enterprise—the presence of numerous representative men in every department of medical art and science, the carefully-prepared programmes for sectional work, the admirably located and easily accessible rooms; while the bountiful hospitality of the London profession, displayed in elegant receptions, banquets, excursions, garden-parties, etc., as well as in private entertainments, afforded both stimulus and opportunity for individual discussions and arguments, from which the real advantage and benefit of such meetings accrue.

The most impressive scene was the opening meeting in St. James' Hall. The floor and gallery were filled with perhaps 3,000 medical men, among whom gray and bald heads predominated. The chair was occupied by one of the most eminent of English physicians—a worthy representative of the immortal name of Jenner. On the right of the chair lounged the good-natured, heavy-jawed, puffy-eyed heir to the throne of England, whose somewhat *blasé* mien presented a marked contrast to the manly features, erect figure and martial bearing of his illustrious brother-in-law and neighbor, the Crown Prince of Germany. Behind these sat a small, spare, rather effeminate looking man, whose mincing movements and careful toilet seemed curiously inappropriate for the famous Langenbeck, of Berlin. A striking contrast was a typical Englishman who sat near—a stout, compact figure, ruddy face, massive head, crowned with long silvery hair, keen gray eyes, thin compressed lips—agile, nervous Spencer Wells. Close by, a modest, rather shabby, insignificant looking individual, with the characteristic sandy complexion of the Scotch, was Keith. At a table on the left, an object of general attention, sat a wiry, restless figure, conspicuous by high cheek-bones, a head tapering like a cocoanut, and a disposition to chatter constantly, the most genial scholar and scholarly genius of them all—Rudolf Virchow.

Further back, a tall, spare, muscular figure, distinguished among the gray heads by coal-black hair, with a rather austere, puritanical countenance (aside from a most unpuritanical blush at the end of the nose), and with a gravity of manner becoming his puritanical name—Jonathan Hutchinson. A massive, rather unwieldy form, with a fine head, sallow skin, and iron-gray hair and beard, arose in response to calls of Pasteur. A most impressive face—delicate features, high forehead, clear eyes and flowing white beard, added to a suavity of manner worthy of a princess' husband, distinguished Esmarch of Kiel. Near him sat a man whose name instantly rivets attention upon a face notable only for the immense expanse of brow. It seems incredible that this quiet, youthful looking individual (he does not seem forty-five) can be Volkmann, of Halle.

But I will not weary you with further enumeration of the hundred eminent men who occupied the same platform.

After a brief and sensible introduction by Sir William Jenner, delivered in a timid, clumsy way, the Prince of Wales read a few platitudes, and declared the Congress open. Then came a most eloquent practical address by the President, Sir James Paget, the delivery of which, in a beautifully clear, well-modulated voice, though marred by occasional awkward gestures, secured the close attention of the vast audience for three-quarters of an hour, after which the Congress adjourned for sectional work.

This work consisted, to an unpleasant degree, of twaddle by ambitious nonentities, who hoped to achieve immortality by appearance on the rostrum of the Congress, and who thereby wasted much valuable time in the reports of wonderful operations, in the description of wonderful apparatus, in the promulgation of wonderful views and theories. Yet several questions elicited real discussion, in which the leaders of medical and surgical practice put upon record their views of several still unsettled topics. It is flattering to our national pride, that we have produced not only the best horses, oarsmen and pedestrians of the year, but that the palm for advances in surgery has been almost unanimously awarded to America. Lithotrity at a single sitting, involving often the use of a large catheter (29 or 30 Charrière) for evacuation, and the presence of instruments in the bladder for even two or three hours—in short, *Bigelow's* operation, received flattering testimonials from many, and marked approval from *all* speakers except Sir Henry Thompson. He objects to the use of the large catheter, which Bigelow insists is the essential feature of his operation, preferring to use a small lithotrite for the removal of the larger fragments. His friends are compelled to admit that, while this procedure may be safe and effectual in Sir Henry's practiced hand, it might be quite different in that of the average surgeon; while his enemies (and they are numerous in London) ascribe his opposition to personal jealousy of Bigelow. The latter's operation, therefore, while it may not effect, as the enthusiastic Telvan affirmed, a revolution in vesical surgery, is certainly the recognized and fashionable mode of lithotrity.

Emmett's operation for lacerated and everted cervix, although meeting far more vigorous opposition than Bigelow's, received the endorsement of all the representatives of the American gynæcological school, and of several among the English. Playfair, in particular, made a terse and witty speech narrating his conversion to Emmett's views, as based upon a case which he (P.) had "tinkered" with various caustics for several years, and which Emmet cured completely by a single operation. The operation will doubtless now receive at least a fair trial by the conservative English, few of whom have hitherto deigned to test it, as I was informed personally by Mr. Wells and Dr. Bantock.

The medical and pathological sections labored for two days to determine the relations of renal disease to general vascular changes. Gull and Sutton attempted to force their "arterio-capillary fibrosis" heresy down the reluctant throats of the Germans; Geo. Johnson harped on muscular hypertrophy, as usual; Rosenstein gave an admirable *résumé* of the German views as to the pathology of the kidney; but when Grainger Stewart hove in sight with his interminable catalogue of imaginary "Bright's diseases," the writer fled. What the issue of the conflict was, I have not heard; but as the combatants were chiefly Gaels and Teutons, it is safe to assume that no conclusion was reached.

The sharpest discussion was that upon antiseptic surgery. Spencer Wells said that he had not used a drainage tube in ovariotomy during the last three years. (I have heard him say that he meditates abandoning the spray). Lawson Tait referred to thirty-six cases of unusual intraperitoneal operations—removal of gall-stone, opening of hydatids of liver, cysts of kidney, abscesses of pelvis, section for Fallopian pregnancy, etc. But one patient died. In only a few cases had he attempted to use Listerian details, which he soon abandoned as cumbrous and impracticable; sometimes positively injurious.

Keith stated that after losing two ovariotomy patients by carbolic acid poisoning, he had renounced the spray, and had since had sixty-five cases without a death.

Savory, while complimenting Lister on the improvement in the sanitary condition of King's College Hospital, referred to the fact that antiseptic surgery, in Lister's own hands, could not show results as good as those by the old plan at St. Bartholomew's.

On the other hand, Esmarch, Volkmann and Czerny related wonderful improvement in the statistics of their operations since they had adopted Lister's method. In the Samaritan Hospital here Thornton had twenty-one cases of ovariotomy, with one death—all carefully Listered; his colleague, Bantock, shows a similar record, except that he will not allow a drop of carbolic acid or other antiseptic to be used during an operation—employing simple water dressing.

But the crowning blow for antiseptic surgery was dealt by Lister himself. Attempting to reply to Keith, he said that he had always advised against the use of the spray in intra-peritoneal operations! That inasmuch as contagion occurred through fingers and instruments as much as by the air, the spray might perhaps be laid aside without much danger; that although he was not ready to take the step, he *might be* in *another year!* It was an aggravated case of "crawfishing." Esmarch, Volkmann & Co. looked decidedly disconcerted. It looks as if the bacteria superstition was about exploded even in Lister's own mind, and that he is simply "prospecting" for a good excuse, under cover of which he may extinguish his absurd and dangerous spray, and base the claims of antiseptic surgery where they belong.

I can not conclude without recording the brilliant success achieved by Dr. Billings' wise and witty paper on Medical Literature. The close attention, signs of frequent approval and appreciation, and generous applause at the close, were handsomely crowned by Sir James Paget's remark from the chair, that if the Congress could show no other evidence of usefulness than the production of that paper, its existence would still be abundantly justified. W. T. BELFIELD.

---

ASPIRATION IN DISTENDED GALL-BLADDER.—The time has come when the internal administration of drugs in cases of distention of the biliary passages should not be exclusively relied upon. Surgical interference should be practiced much more frequently than has been done. The advantages which aspiration possesses over other means of emptying the dilated gall-bladder, may be thus formulated:

I. The operation may be performed with *safety*, without taking particular precautions in uniting the walls of the gall-bladder with those of the abdomen.

II. The operation can therefore be done as soon as the diagnosis of the dilated gall-bladder has been made, if, from its size, there seems to be danger of rupture; or if the patient suffers much pain. Aside from these conditions, when aspiration should be resorted to without hesitation, the question presents itself whether it would not be good practice to evacuate the contents of a distended gall-bladder under all circumstances, simply to remove the superfluous bile, which, being cut off from its natural destination, is bound to be reabsorbed by the lymphatics, carried back into the circulation and produce, to a greater or lesser degree, a condition which is generally known as "cholæmia."

III. A very fine trocar, such as would be of not much value in case of simple puncture, can be employed, and by means of suction even a tenacious fluid can be removed from the gall-bladder.

IV. The insertion of a trocar or an aspirating needle is almost a painless procedure.

V. In cases of doubt as to the presence of gall-stones, a flexible probe can be passed through the canula and used as a sound.

VI. Aspiration being a safe and painless operation, it can be employed for the purpose of aiding diagnosis.—*Kretzschmar.*

